# Body composition in male elite athletes, comparison of bioelectrical impedance spectroscopy with dual energy X-ray absorptiometry

**DOI:** 10.1186/1477-5751-7-1

**Published:** 2008-01-22

**Authors:** Ulla Svantesson, Martina Zander, Sofia Klingberg, Frode Slinde

**Affiliations:** 1 Institute of Neuroscience and Physiology, Sahlgrenska Academy, University of Gothenburg, Sweden; 2 Department of Clinical Nutrition, Sahlgrenska Academy, University of Gothenburg, Sweden; 3 School of Life Sciences, University of Skövde, Skövde, Sweden

## Abstract

**Background:**

The aim of this study was to compare body composition results from bioelectrical spectroscopy (BIS) with results from dual energy X-ray absorptiometry (DXA) in a population of male elite athletes. Body composition was assessed using DXA (Lunar Prodigy, GE Lunar Corp., Madison, USA) and BIS (Hydra 4200, Xitron Technologies Inc, San Diego, California, USA) at the same occasion. Agreement between methods was assessed using paired t-tests and agreement-plots.

**Results:**

Thirty-three male elite athletes (soccer and ice hockey) were included in the study. The results showed that BIS underestimates the proportion of fat mass by 4.6% points in the ice hockey players. In soccer players the BIS resulted in a lower mean fat mass by 1.1% points. Agreement between the methods at the individual level was highly variable.

**Conclusion:**

Body composition results assessed by BIS in elite athletes should be interpreted with caution, especially in individual subjects. BIS may present values of fat mass that is either higher or lower than fat mass assessed by DXA, independent of true fat content of the individual.

## Background

In many sports, the body composition of the individual athlete plays an important role. Changes in body composition might be a marker of change in nutritional status. Changes in body composition have been used as information regarding the athlete's adaptation to different types of training [1]. It has been shown that a high proportion of body fat mass (FM) is related to a low power to weight-ratio, reduced acceleration and increased energy expenditure, while the opposite applies to a high proportion of fat free mass (FFM) [2]. On the other hand, a low proportion of body fat has also been shown to reduce performance [3]. The optimal body composition varies between sports; in precision sports such as golf, bowling and shooting, the results are less dependent upon body composition as in sports as soccer, gymnastics and figure skating [4]. Sudden changes in body composition can be a sign of health problems, the most known being the female athlete triad [5]. It therefore seems crucial that coaches and other leaders within sports have knowledge and equipment to assess body composition. Such equipment should be cheap, easy to transport, give reliable results, and should not require large education effort. Such a method could be bioelectric impedance spectroscopy (BIS).

Our understanding of the composition of the human body is based on chemical analysis of six human bodies [6-9]. These analyses showed that the mean water content of the human FFM is 724 g per kg FFM. This finding has been confirmed in 50 guinea pigs [10]. Bioelectrical impedance assessment (BIA) is one method to achieve an estimation of total body water (TBW). BIA makes use of the fact that impedance to electrical flow of an injected current is related to the volume of the conductor (the human body) and the square of the conductor's length (height). Impedance is a measure of how electrical current is lowed or stopped as it passes through a material. Thomasset [11] was the first to report a relation between body water and electrical impedance. Hoffer et al. [12] developed the principle and demonstrated that total body water determined by the tritiated water method was strongly correlated (r = 0.92) with height^2^/impedance in 20 normal volunteers and 34 patients with varying diagnosis and hydration status. Since then, numerous validation studies have been published. Some validation studies of BIA using linear models have been performed in athletes, and most of these report good validity, but only on group level [3,13-15]. BIA has been a widely adopted method for body composition assessment, not only for scientific purposes but also in clinics and leisure centres [16].

A common used reference method for body composition assessment is dual energy X-ray absorptiometry (DXA) which also has been used in studies of athletes showing high reproducibility [3,13]. DXA was originally developed to examine bone mineral density and examines the body in mm^3 ^dividing the human body in three parts: bone, fat- and bone free (soft) tissue, and fat tissue. The European Society for Clinical Nutrition and Metabolism recommends DXA as reference method in body composition studies [17].

BIS is one of the latest technical developments within this area. It differs from the older methods by measuring impedance using a spectrum of frequencies and calculates body composition using non-linear mathematical models [16]. BIS has not yet been explored in athletes. A method to be used in the world of sports should also be valid on the individual level. The aim of this study was therefore to compare body composition results from bioelectrical spectroscopy with results from dual energy X-ray absorptiometry (DXA) in a population of male elite athletes.

## Results

Characteristics of the different groups of athletes are presented in Table 1. The ice hockey players had statistical significant higher body weight and BMI, compared to the soccer players. All ice hockey players but five had a BMI > 25 kg/m^2^. Table 2 shows that BIS overestimate the amount of fat-free mass and underestimate the amount of fat mass, compared to the result from DXA. This is especially obvious among the ice hockey players showing a statistically significant higher fat free mass, assessed by BIS, compared to the soccer players. All participants had a body fat content assessed by DXA < 20% of their body weight.

**Table 1:** Characteristics of study subjects (mean (sd)).

	All participants (n = 33)	Ice hockey players (n = 16)	Soccer players (n = 17)
Age (y)	24.8 (5.0)	25.6 (6.1)	24.1 (3.8)
Body weight (kg)	83.3 (7.2)	86.3 (5.3)	80.6 (7.7)*
Body height (cm)	183.6 (5.7)	183.7 (5.0)	183.5 (6.4)
BMI (kg/m^2^)	24.7 (1.5)	25.6 (1.2)	23.9 (1.3)*

* p < 0.05, unpaired t-test

**Table 2:** Body composition results (mean (sd)).

	All participants (n = 33)	Ice hockey players (n = 16)	Soccer players (n = 17)
Fat free mass from DXA (kg)	73.8 (5.2)	75.4 (3.4)	72.4 (6.2)
Fat free mass from BIS (kg)	75.8 (7.1)	78.9 (4.6)	72.8 (7.9)^†^
p*	0.0056	0.00069	0.64
Fat mass from DXA (% of body weight)	11.9 (3.8)	13.0 (4.0)	10.9 (3.5)
Fat mass from BIS (% of body weight)	9.1 (3.9)	8.4 (4.2)	9.7 (3.6)
p*	0.00074	0.00022	0.28

* paired t-test, DXA compared to BIS

^† ^p < 0.05, soccer players compared to ice hockey players (unpaired t-test)

**Figure 1:**
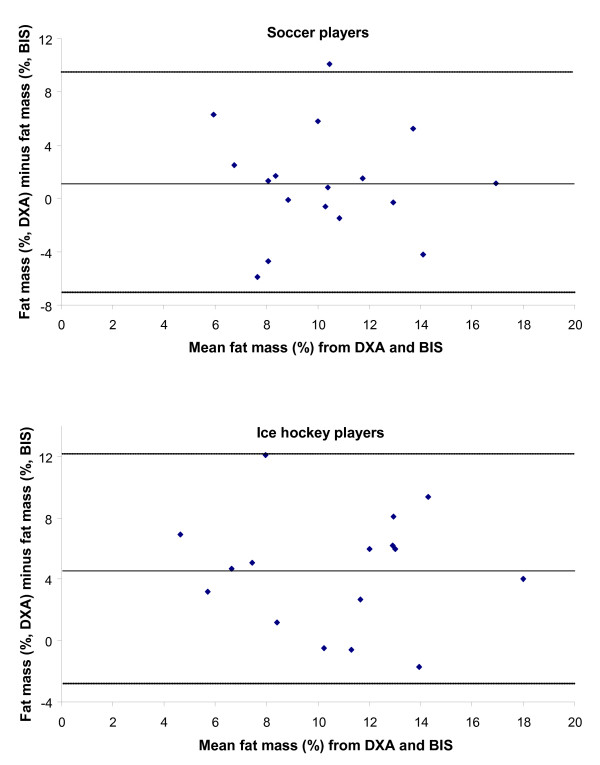
Differences between proportion of fat mass assessed by BIS and DXA plotted against average of proportion of fat mass assessed by BIS and DXA in 17 male elite soccer players and 16 male elite ice hockey players. Lines indicates mean ± 2 SD.

The Bland-Altman plots presented in Figure 1 shows that BIS underestimates the proportion of fat mass by 4.6% points in the ice hockey players. In soccer players the BIS resulted in a lower mean fat mass by 1.1% points. Agreement between the methods at the individual level is highly variable with the largest difference between methods seen in a male ice hockey player where BIS underestimated the proportion of fat mass by 12.1% points. The largest individual overestimation of fat mass by BIS was found in a soccer player having a fat mass of 5% assessed by DXA and 11% assessed by BIS, a difference of 6% points.

## Discussion

This study shows that BIS underestimates fat mass in a group of male elite athletes compared to results from DXA. It also seems that BIS has very low precision in estimating body composition at an individual level, which could be important information regarding the athlete's adaptation to different types of training [1]. To our knowledge, this is the first study reporting the validity of BIS in elite athletes, and the technique of bioelectrical impedance overall at an individual level in elite athletes. Fornetti et al [3] found good agreement on group level between BIA and DXA in a large sample of female athletes from mixed sports. That was also the case in a study of female dancers [15] and a study of female runners [14]. In these two studies, new prediction equations from BIA, based on regression, were developed since the standard equations did not perform well in the sample of athletes. The choice of prediction equation has also been shown to be of importance in elderly subjects [18]. We chose to use the equation provided by the manufacturer of the BIS equipment, since this probably would have been the fact in daily practice within training facilities. New prediction equations based on the BIS results were not been developed in the present study due to a small sample size. BIS is also a more complex method, compared to BIA, resulting in a large amount of measuring variables, why an equation derived from the current study would probably not be a practical tool to be used out in the field – even though a better validity could have been accomplished.

BIS showed better agreement with DXA in soccer players than in ice hockey players. Segmental data from the DXA showed that the ice-hockey players had larger arms compared to the soccer players, both fat mass, fat free mass and total mass (results not shown). This is not surprising considering the nature of the sports. There were no differences in trunk and leg mass. This might affect the impedance as the volume of the narrowest conductor (the arm) was larger in the ice-hockey players. More important for the differences between the two groups is probably the fact that the soccer players had a more "normal" body which might increase the probability for a prediction equation based on a normal population to provide more valid results.

In this study, DXA was used as reference method. Criticism has even been raised concerning this method and some studies have reported imprecision in body composition assessment [19-22]. A large problem seems to be diversities between different devices and software. However, in this study, only one type of device and software has been used. Even if, to our knowledge, DXA has not been validated in a population of elite athletes, the imprecision shown in other populations have been small and DXA is recommended as reference method in body composition assessment by The European Society for Clinical Nutrition and Metabolism [17].

Results from the current study do not indicate that BIS under- or overestimates fat mass in a systematic pattern. The over- or underestimations do not follow the differences in body fat content of the individual (Figure 1). Skin temperature, strenuous exercise, dehydration, and glycogen depletion have been shown to affect results from measurements of bioelectrical impedance [23,24]. All these are factors commonly appearing after exercise. All participants in the current study were measured under uncontrolled conditions. This might be a reason for the large individual variation in difference between methods and the main limitation with this study is that the participants' conditions with regard to exercise, dehydration or fasting were not registered. However, since all measurements were performed in a narrow time limit (between 1 and 3 PM) this factor is less likely to be the main explanation to the lack of consistency of the results.

In conclusion, BIS may present values of fat mass that is either higher or lower than fat mass assessed by DXA, independent of true fat content of the individual.

## Conclusion

The optimal body composition varies between different sports. It seems crucial that coaches and other leaders within sports have knowledge and equipment to assess body composition. Such equipment should be cheap, easy to transport, give reliable results, and should not require large education effort. Such a method could be bioelectric impedance spectroscopy (BIS). This study shows that body composition results assessed by BIS in male elite athletes should be interpreted with caution, especially in individual subjects, which may be the main use of this assessment method. Especially overestimation of the fat mass with BIS might have serious health hazards if interpreted in the wrong way.

## Methods

Adult male athletes were recruited during spring 2006 – spring 2007 from athletic clubs in the Göteborg region, Western Sweden. All athletes were 18 years or older and competed in the highest Swedish league in their individual sport. The subjects included in this study were participating in soccer and ice hockey. All athletes received oral and written information concerning the study before they gave their written consent. The Regional Ethical Review Board in Göteborg, Sweden approved the study protocol.

All measurements were performed in the afternoon (between 1–3 PM) at the same occasion for each athlete. Body weight was measured, with subjects wearing underwear, to the nearest 0.1 kg on a System 31 electronic scale (The Advanced Weighing Co. Ltd, New Haven, East Sussex, UK). Height was measured and determined to the nearest centimetre using a horizontal headboard with an attached wall-mounted metric rule (Hultafors, Sweden). BMI was calculated as weight (kg) divided by height^2 ^(m).

Body composition was measured with DXA (Lunar Prodigy, GE Lunar Corp., Madison, USA) and BIS (Hydra 4200, Xitron Technologies Inc, San Diego, California, USA). Precision of the DXA equipment was estimated from nine repeated measurements with coefficients of variation of body fat percentage of 2.1%. Two BIS measurements were taken on the right side of the body and a mean value of the results were used as each athletes' result. The BIS measurements were taken after the athlete had been in a supine position for 10 min. The electrodes (Red Dot surveillance electrode (2239) for single use with foam tape and sticky gel Ag/AgCl (3 M)) were positioned, at the middle of the dorsal surfaces of the hand and feet, respectively, proximally to the metacarpal-phalangeal and metatarsal-phalangeal joints and medially between the distal prominence of the radius and the ulna and between the medial and lateral malleoli of the ankle joint. Body composition was calculated using equations provided by the manufacturer. Precision of the BIS equipment was estimated from the two measurements in the current study with coefficients of variation of fat free mass of 0.3%.

Results are described as mean and standard deviation (sd). Differences between sports were tested using unpaired t-test. The statistical method described by Bland and Altman [25] was used to assess the degree of agreement between body composition assessed by DXA and body composition assessed by BIS. Differences between methods were also tested using paired t-test. Level of statistical significance was set to 0.05. All statistical analyzes were performed in SPSS 13.0 for Windows (SPSS Inc, Chicago, USA).

## Authors' contributions

US conceived of the study, participated in its design and helped to draft the manuscript. MZ performed the bioelectrical impedance spectroscopy measurements, analysed the data and helped to draft the manuscript. SK participated in the study design and coordination and helped to draft the manuscript. FS coordinated the data collection, performed the statistical analysis and drafted the manuscript.
